# Modeling and Imaging of Ultrasonic Array Inspection of Side Drilled Holes in Layered Anisotropic Media

**DOI:** 10.3390/s21144640

**Published:** 2021-07-06

**Authors:** Chirag Anand, Roger M. Groves, Rinze Benedictus

**Affiliations:** 1 Structural Integrity and Composites Group, Faculty of Aerospace Engineering, Delft University of Technology, 2629 HS Delft, The Netherlands; R.M.Groves@tudelft.nl (R.M.G.); R.Benedictus@tudelft.nl (R.B.); 2 Aerospace Non-Destructive Testing Laboratory, Faculty of Aerospace Engineering, Delft University of Technology, 2629 HS Delft, The Netherlands

**Keywords:** Gaussian beam, full matrix capture, total focusing method, ultrasonic phased array, CFRP, side-drilled hole

## Abstract

There has been an increase in the use of ultrasonic arrays for the detection of defects in composite structures used in the aerospace industry. The response of a defect embedded in such a medium is influenced by the inherent anisotropy of the bounding medium and the layering of the bounding medium and hence poses challenges for the interpretation of the full matrix capture (FMC) results. Modeling techniques can be used to understand and simulate the effect of the structure and the defect on the received signals. Existing modeling techniques, such as finite element methods (FEM), finite difference time domain (FDTD), and analytical solutions, are computationally inefficient or are singularly used for structures with complex geometries. In this paper, we develop a novel model based on the Gaussian-based recursive stiffness matrix approach to model the scattering from a side-drilled hole embedded in an anisotropic layered medium. The paper provides a novel method to calculate the transmission and reflection coefficients of plane waves traveling from a layered anisotropic medium into a semi-infinite anisotropic medium by combining the transfer matrix and stiffness matrix methods. The novelty of the paper is the developed model using Gaussian beams to simulate the scattering from a Side Drilled Hole (SDH) embedded in a multilayered composite laminate, which can be used in both immersion and contact setups. We describe a method to combine the scattering from defects with the model to simulate the response of a layered structure and to simulate the full matrix capture (FMC) signals that are received from an SDH embedded in a layered medium. The model-assisted correction total focusing method (MAC-TFM) imaging is used to image both the simulated and experimental results. The proposed method has been validated for both isotropic and anisotropic media by a qualitative and quantitative comparison with experimentally determined signals. The method proposed in this paper is modular, computationally inexpensive, and is in good agreement with experimentally determined signals, and it enables us to understand the effects of various parameters on the scattering of a defect embedded in a layered anisotropic medium.

## 1. Introduction

In recent years, there has been an increase in the usage of composites in aircraft structures, such as for the Boeing 787 and the Airbus A350 [1]. A variety of methods are used to test such structures, but ultrasonic inspection is the most widely used due to its sensitivity to defects present in composite structures, its ability to localize such defects, and its speed of detection [2,3]. There has been a rapid increase in the ultrasonic phased array testing of composite structures used in the aerospace industry [4]. The testing of such structures is complicated due to the inherent anisotropy of composites and the presence of multiple layers. The purpose of non-destructive inspection of structures is to detect and locate defects that are present in them, such as cracks, voids, delaminations, and disbonds [2,5,6,7]. The output ultrasonic signal from defects in such structures is influenced by the anisotropy and layering present in them. Hence, computational models are required that take the anisotropy, layering, and the response of the defect into account. 

A variety of approaches to simulate the array signals from multilayered materials have been reported in the literature. These approaches include applying ray methods to a homogenized layered structure [8], using hybrid ray–finite difference time domain (FDTD) methods [9], multi-Gaussian beams [10,11], or using plane wave models to calculate the reflection or transmission of the waves in the bounding media [12,13,14,15]. These approaches are either computationally expensive when used for layered materials, singular when interacting with curved interfaces, or do not reflect the real-world situation of bounded beams. Anand et al. [16] used Gaussian beams due to their computational efficiency and non-singularity when interacting with curved interfaces, combined with the recursive stiffness matrix method, which enables the response of the layered structure to be taken into account, when modeling the bounded beam interaction between phased arrays and multilayered media. One of the preliminary assumptions of the model was that the multilayered laminate is bounded by a semi-infinite water layer, allowing only longitudinal waves to impinge onto the composite laminate and thereby reducing the number of unknowns [17]. 

To simulate the scattering response of defects embedded within the laminate, traditionally, finite element methods (FEM) [18] have been used, which are computationally expensive as the inspection frequency, number of elements used in the array, and number of layers in the laminate increase. For the simulation of defects of simple shapes such as side-drilled holes, an analytical model is computationally less expensive [19]. To the best of the authors’ knowledge, the use of Gaussian beams to simulate such an interaction with defects embedded in a multilayered material does not exist. The model developed by Anand et al. [16] is based on the assumption of water bounding layers, which is invalid as the defect is surrounded by a homogeneous isotropic or anisotropic elastic medium. To address this limitation, in this paper, we provide a method to calculate the reflection and transmission coefficients for a multilayered laminate bounded by a semi-infinite anisotropic medium. For a layered structure such as a quasi-isotropic carbon fiber-reinforced plastic (CFRP) laminate, which has a repeated set of layers of different orientations, the lower bounded medium of the embedded defect can be modeled as an equivalent homogeneous anisotropic medium, as the dominating signal is the scattering from the defect and the reflections from the plies below it can be neglected [20].

Defects such as side-drilled holes (SDH) are commonly used as reference defects for ultrasonic phased array testing [21]. This is because SDH also gives rise to the various wave–defect interactions, such as scattering, creeping waves, change in wave mode, etc., which can be observed with commonly encountered defects in metallic and composite structures. In this paper, we develop a model to simulate the scattering from an SDH that is embedded in a layered CFRP laminate. The model simulates the received full matrix capture (FMC) signals from the scattering of an SDH, and the modified total focusing method (TFM) algorithm is used to image the defect from FMC signals generated from simulations and experimentally. The novelty of this paper is that it provides an analytical modeling technique to model and simulate the responses of defects that are embedded in layered anisotropic structures such as composite structures. The analytical model takes into account the various effects of anisotropy and layering on the received signal. The analytical model is computationally inexpensive. The paper also provides a model-assisted correction total focusing method imaging algorithm to image defects in anisotropic structures. The next section provides the background theory used for modeling the scattering from an SDH in a layered anisotropic medium.

## 2. Background Theory

The following sections give a brief description of the stiffness matrix and transfer-matrix methods. An understanding of the transfer matrix method is required as it forms the basis for the matrix formulation of the transmission/reflection of plane waves from a layered medium from/into a generally anisotropic semi-infinite medium. It is then followed by the theory for Gaussian beam modeling of transducers and for calculating the equivalent homogeneous properties of a layered medium. This section ends with the theory of scattering from an SDH.

### 2.1. Transfer and Stiffness Matrix Method for Multilayer Wave Propagation

**Figure 1:**
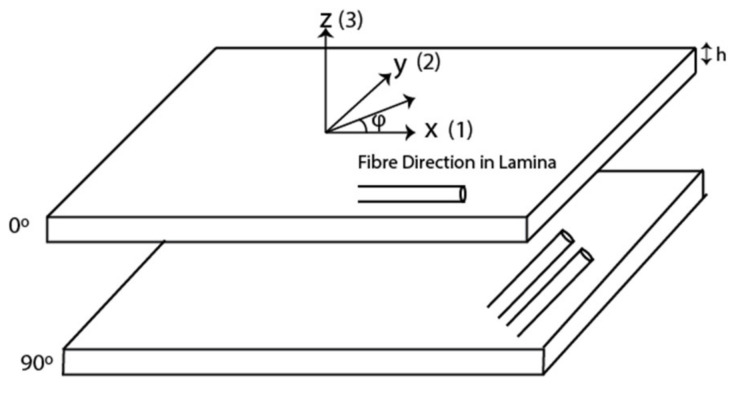
CFRP laminas specifying the local axis.

We consider a multilayered CFRP laminate as seen in Figure 1.

The laminate is composed of N number of layers, which are homogeneous and anisotropic. The layers are of thickness *h* and are of infinite extent in the plane of the lamina (*x* − *y*). The laminate is bounded by an upper and lower bounding medium, m = 0 and m = N + 1, respectively. A plane wave strikes the top surface of the laminate at an incident angle of θ with respect to the z axis. The projection of the wave vector on the x–y plane is denoted by φ. For ease and simplicity, the local coordinates are denoted by the numbers 1, 2, and 3, respectively and hence the coordinates are *x*_1_*, x*_2_, and *x_3_*. Hence, the displacement of a plane wave in a layer is given by Equation (1):

(1) u=exp(i(kx−ωt))

where **k** is the wavenumber vector consisting of *k*_1_, *k*_2_, and *k*_3_ components, ω is the angular frequency, and *t* is the time. The wavenumber components *k*_1_ and *k*_2_, which lie in the plane of the laminate, remain unchanged, due to Snell’s law, whereas the wavenumber component *k*_3_ undergoes a change. The wavenumber component *k*_3_ can be calculated using the Christoffel equation [22] as shown below:

(2) $$\left(c_{ijkl}k_{j}k_{k}-\rho \omega^{2}\partial_{il}\right)d_{l}=0$$

where *c_ijkl_* is the stiffness tensor, *ρ* is the density of the material, *δ_il_* is the Kronecker delta, *d_l_* is the polarization vector component for different wave modes—quasi-longitudinal, quasi-shear horizontal, and quasi-shear vertical—and *i, j, k, l* consist of values 1, 2, 3 corresponding to the three axes *x*, *y*, *z*. *k*_3_ can be obtained by solving the Christoffel equation shown in Equation (2) and will consist of two solutions for each propagating wave mode. One solution corresponds to the downward traveling wave in the layer and the other corresponds to the upward traveling wave, denoted by ‘+’ and ‘−’, respectively. The wave modes are represented by *p*, with values 1, 2, and 3 for quasi-shear horizontal, quasi-shear vertical, and quasi-longitudinal waves, respectively.

Hence, the plane wave displacement in the layer *m* can be calculated as shown below [23] by considering the wave amplitudes and the wavenumber components:

(3) $$u_{i}^{m}=\sum_{p=1}^{3}a^{m,p+}d_{i}^{m,p+}e^{ik_{3}^{m,p}x_{3}^{m,p}}+a^{m,p-}d_{i}^{m,p-}e^{-ik_{3}^{m,p}x_{3}^{m,p}}\times e^{i\left(k_{1}x_{1}+k_{2}x_{2}-\omega t\right)}$$

where $a^{m,p+/-}$ are the wave amplitudes of the downward and upward traveling waves of mode *p* in the layer *m*. The *x_3_* coordinates are the local coordinates of the layer *m.* The relationship between the stress and displacement in the layer is given by

(4) $$\sigma_{ij}=\frac{1}{2}c_{ijkl}\left(\frac{\partial u_{k}}{\partial x_{l}}-\frac{\partial u_{l}}{\partial x_{k}}\right)$$

The next step is to calculate the transfer matrix for the layer *m.* Substituting Equation (3) into Equation (4) and rearranging the displacement and stress at the top surface of the layer *m* gives Equation (5):

(5) $$\left[\begin{array}{l} u^{m}\left(0\right)\\ \sigma^{m}\left(0\right) \end{array}\right]=\left[\begin{array}{cc} D^{+} & D^{-}H^{-}\\ F^{+} & F^{-}H^{-} \end{array}\right]\left[\begin{array}{l} A^{m+}\\ A^{m-} \end{array}\right]$$

and at the bottom surface of layer *m* by Equation (6)

(6) $$\left[\begin{array}{l} u^{m}\left(h_{m}\right)\\ \sigma^{m}\left(h_{m}\right) \end{array}\right]=\left[\begin{array}{cc} D^{+}H^{+} & D^{-}\\ F^{+}H^{+} & F^{-} \end{array}\right]\left[\begin{array}{l} A^{m+}\\ A^{m-} \end{array}\right]$$

where **u^m^** and **σ^m^** are the displacement and stress matrices for layer m, **A^m±^** are the amplitudes of the upward traveling waves in layer m, *h^m^* is the thickness of the layer m, **F** is the matrix consisting of force vectors **f^±^** of the three propagating modes of the wave in Equation (7), D is a matrix consisting of the polarization vectors as shown in Equation (8), and **H** is a diagonal matrix in which the propagators are distributed along the diagonal with the other elements of matrix being zero, as shown in Equation (9).

(7) $$\begin{array}{l} F^{\pm }=[\begin{array}{ccc} f^{\pm 1} & f^{\pm 2} & f^{\pm 3} \end{array}]\\ f^{\pm p}{}_{i}=i(c_{i3kl}k_{l}d_{k}) \end{array}$$

(8) $$D^{\pm }=[\begin{array}{ccc} d^{\pm 1} & d^{\pm 2} & d^{\pm 3} \end{array}]$$

(9) $$H^{\pm }=Diag[\begin{array}{ccc} e^{ik_{3}^{\pm 1}h_{m}} & e^{ik_{3}^{-2}h_{m}} & e^{ik_{3}^{-3}h_{m}} \end{array}]$$

In Equations (7)–(9), subscripts 1, 2, and 3 correspond to the different wave modes. In the transfer matrix method, we rearrange to obtain

(10) $$\left[\begin{array}{l} u^{m}\left(0\right)\\ \sigma^{m}\left(0\right) \end{array}\right]=B_{m}\left[\begin{array}{l} u^{m}\left(h_{m}\right)\\ \sigma^{m}\left(h_{m}\right) \end{array}\right]$$

(11) $$B_{m}=\left[\begin{array}{cc} D^{+}H^{+} & D^{-}\\ F^{+}H^{+} & F^{-} \end{array}\right]{\left[\begin{array}{cc} D^{+} & D^{-}H^{-}\\ F^{+} & F^{-}H^{-} \end{array}\right]}^{-1}$$

where the stress and displacement on the top of layer m are related to the stress and displacement at the bottom of layer m by a transfer matrix **B***_m_*.

In order to define the transfer matrix of the entire structure, continuity of stress and displacement constraints are applied at each layer interface. Hence, the transfer matrix **B** for the entire structure can be determined:

(12) $$\left[\begin{array}{l} u^{0}\left(0\right)\\ \sigma^{0}\left(0\right) \end{array}\right]=B\left[\begin{array}{l} u^{n}\left(h_{N}\right)\\ \sigma^{n}\left(h_{N}\right) \end{array}\right]$$

Similarly, in the stiffness matrix method, we obtain an equation where the stresses on the top and bottom of layer *m* are related to the displacement at the top and bottom of layer *m* by a stiffness matrix **S***_m_*.

(13) $$\left[\begin{array}{l} \sigma^{m}\left(0\right)\\ \sigma^{m}\left(h_{m}\right) \end{array}\right]=S_{m}\left[\begin{array}{l} u^{m}\left(0\right)\\ u^{m}\left(h_{m}\right) \end{array}\right]$$

where

(14) $$S_{m}=\left[\begin{array}{cc} F^{+} & D^{-}H^{-}\\ F^{+}H^{+} & F^{-} \end{array}\right]{\left[\begin{array}{cc} D^{+} & D^{-}H^{-}\\ D^{+}H^{+} & D^{-} \end{array}\right]}^{-1}$$

Similar to the method used to determine **B**, the stiffness matrix **S** relating the stress in the upper semi-infinite bounding layer and the lower semi-infinite bounding layer to the respective displacements can also be determined by applying continuity of stress and displacement constraints:

(15) $$\left[\begin{array}{l} \sigma^{0}\left(0\right)\\ \sigma^{N}\left(h_{N}\right) \end{array}\right]=S\left[\begin{array}{l} u^{0}\left(0\right)\\ u^{N}\left(h_{N}\right) \end{array}\right]$$

The next section shows the theoretical fundamentals of multi-Gaussian beams.

### 2.2. Modeling of the Transducer Gaussian Beams

Multi-Gaussian beams [24] can be used to model the radiation from phased array transducers by superimposition of Gaussian beams with different Wen and Breazeale coefficients. Hence, the velocity at a distance *x*_1_ is calculated as shown below:

(16) $$\begin{array}{l} v_{e}\left(x_{1},\omega \right)=d\exp\left(i\omega \frac{x_{3}}{c}\right)\\ \sum_{o=1}^{10}\sum_{q=1}^{10}\begin{array}{l} \frac{A_{o}A_{q}}{{\sqrt{1+cx_{3}\left[M_{oq}(0)\right]}}_{11}{\sqrt{1+cx_{3}\left[M_{oq}(0)\right]}}_{22}}\\ \exp\left[\frac{1}{2}X^{T}M_{oq}(x_{3})X\right] \end{array} \end{array}$$

where ***X*** represents the coordinates between the *e*^th^ transmitting element and the receiving element, *c* is the wave velocity, *x*_3_ is the distance traveled along the *z* axis in Figure 1, **d** is the polarization vector, and *o* and *q* have values ranging from 1 to 10, which correspond to the ten Wen and Breazeale coefficients.

(17) $$\begin{array}{l} {\left[M_{oq}(0)\right]}_{11}=\frac{iB_{o}}{R_{1}},{\left[M_{oq}(0)\right]}_{22}=\frac{iB_{q}}{R_{2}}\\ R_{1}=\frac{ka_{1}{}^{2}}{2},R_{2}=\frac{ka_{2}{}^{2}}{2} \end{array}$$

(18) $$\begin{array}{l} {\left[M_{oq}(x_{3})\right]}_{11}=\frac{{\left[M_{oq}(0)\right]}_{11}}{1+cx_{3}{\left[M_{oq}(0)\right]}_{11}}\\ {\left[M_{oq}(x_{3})\right]}_{22}=\frac{{\left[M_{oq}(0)\right]}_{22}}{1+cx_{3}{\left[M_{oq}(0)\right]}_{22}}\\ {\left[M_{oq}(x_{3})\right]}_{12}={\left[M_{oq}(x_{3})\right]}_{21}=0 \end{array}$$

In the above equations, *k* is the wavenumber in the direction of propagation of the wave, and *a*_1_ and *a*_2_ are the width and length of the rectangular transducer, respectively. *A_o_*, *A_q_*, *B_o_*, *B_q_* are the Wen and Breazzle coefficients [25].

Hence, at the face of the transducer, where x_3_ = 0, the velocity distribution is given below:

(19) $$v_{e}\left(x,\omega \right)=\sum_{o=1}^{10}\sum_{q=1}^{10}A_{o}A_{q}\exp\left[\frac{1}{2}X^{T}M_{oq}(x_{3})X\right]$$

The velocity distribution in the wavenumber–frequency domain can be calculated as given below:

(20) $$v_{e}\left(k,\omega \right)=\int_{-\infty }^{\infty }v_{e}\left(x,\omega \right)e^{-ikx_{1}}dx_{1}^{}$$

The velocity distribution obtained in the wavenumber–frequency domain will be used in Section 3.2 to calculate the received signal from the scattering from an SDH.

### 2.3. Equivalent Homogeneous Anisotropic Properties of a Thick Laminate

When a layered composite laminate such as CFRP with repeated layers is tested at lower frequencies, i.e., longer wavelengths, where the thickness of the plies is less than the wavelength of the wave, the reflections from the ply interfaces are negligible and have no effect on the propagation of the wave [26]. In such a scenario, the laminate can be considered to have equivalent homogenous properties, which can be used for calculating the group velocity of the laminate and for imaging purposes, etc. Many methods have been investigated to calculate the equivalent homogeneous properties. For this paper, the method described by Sun and Li [20] is chosen as it gives explicit relations to find the homogeneous anisotropic properties.

Classical laminate theory is used for characterizing thin laminates [27]. For thick laminates, higher-order plate theories are used, which are more mathematically complex [28]. In thick laminates with periodic stacking layers, where the characteristic length of deformation of the laminate is larger than the periodicity, the non-homogeneous properties over each typical cell can be replaced by effective properties [29]. Thus, each cell of a laminate can be represented as a homogeneous anisotropic solid. Sun and Li considered a thick laminate consisting of repeated sub-laminates, where the thickness of the sub-laminates was small compared to the thickness of the entire laminate. The sub-laminate was then evaluated using constant stress and strain assumptions, and the effective homogeneous properties of the entire laminate were calculated. The SDH is assumed to be embedded in an anisotropic homogeneous medium as the specular reflection from the defect after the wave has traveled through the upper layered medium needs to be calculated and hence the embedding medium is considered homogeneous. The explicit expressions to calculate the effective homogeneous properties are given below, where *C* is the stiffness tensor in Voigt notation [20]:

(21) $$\bar{C_{11}}=\sum_{m=1}^{N}h_{m}C_{11}^{m}+\sum_{m=2}^{N}h_{m}\left(C_{13}^{m}-\lambda_{13}\right)\left(C_{13}^{1}-C_{13}^{m}\right)/C_{33}^{m}$$

(22) $$\bar{C_{12}}=\sum_{m=1}^{N}h_{m}C_{12}^{m}+\sum_{m=2}^{N}h_{m}\left(C_{13}^{m}-\lambda_{13}\right)\left(C_{23}^{1}-C_{23}^{m}\right)/C_{33}^{m}$$

(23) $$\bar{C_{13}}=\sum_{m=1}^{N}h_{m}C_{13}^{m}+\sum_{m=2}^{N}h_{m}\left(C_{33}^{m}-\lambda_{33}\right)\left(C_{13}^{1}-C_{13}^{m}\right)/C_{33}^{m}$$

(24) $$\bar{C_{22}}=\sum_{m=1}^{N}h_{m}C_{22}^{m}+\sum_{m=2}^{N}h_{m}\left(C_{23}^{m}-\lambda_{23}\right)\left(C_{23}^{1}-C_{23}^{m}\right)/C_{33}^{m}$$

(25) $$\bar{C_{23}}=\sum_{m=1}^{N}h_{m}C_{23}^{m}+\sum_{m=2}^{N}h_{m}\left(C_{33}^{m}-\lambda_{33}\right)\left(C_{23}^{1}-C_{23}^{m}\right)/C_{33}^{m}$$

(26) $$\bar{C_{33}}=1/\sum_{m=1}^{N}h_{m}/C_{33}^{m}$$

(27) $$\bar{C_{16}}=\sum_{m=1}^{N}h_{m}C_{16}^{m}+\sum_{m=2}^{N}h_{m}\left(C_{13}^{m}-\lambda_{13}\right)\left(C_{36}^{1}-C_{36}^{m}\right)/C_{33}^{m}$$

(28) $$\bar{C_{26}}=\sum_{m=1}^{N}h_{m}C_{26}^{m}+\sum_{m=2}^{N}h_{m}\left(C_{23}^{m}-\lambda_{23}\right)\left(C_{36}^{1}-C_{36}^{m}\right)/C_{33}^{m}$$

(29) $$\bar{C_{36}}=\sum_{m=1}^{N}h_{m}C_{36}^{m}+\sum_{m=2}^{N}h_{m}\left(C_{33}^{m}-\lambda_{33}\right)\left(C_{36}^{1}-C_{36}^{m}\right)/C_{33}^{m}$$

(30) $$\bar{C_{66}}=\sum_{m=1}^{N}h_{m}C_{66}^{m}+\sum_{m=2}^{N}h_{m}\left(C_{36}^{m}-\lambda_{36}\right)\left(C_{36}^{1}-C_{36}^{m}\right)/C_{33}^{m}$$

(31) $$\bar{C_{44}}=\left(\sum_{m=1}^{N}h_{m}C_{44}^{m}/\Delta_{m}\right)/\Delta$$

(32) $$\bar{C_{45}}=\left(\sum_{m=1}^{N}h_{m}C_{45}^{m}/\Delta_{m}\right)/\Delta$$

(33) $$\bar{C_{55}}=\left(\sum_{m=1}^{N}h_{m}C_{55}^{m}/\Delta_{m}\right)/\Delta$$

where *h_m_* is the thickness of the ply and

(34) $$\lambda_{13}=\bar{C_{13}},\;\lambda_{23}=\bar{C_{23}},\;\lambda_{33}=\bar{C_{33},}\;\lambda_{36}=\bar{C_{36}}$$

(35) $$\Delta =\left(\sum_{k=1}^{N}\frac{h_{m}C_{44}{}^{m}}{\Delta_{m}}\right)\left(\sum_{k=1}^{N}\frac{h_{m}C_{55}{}^{m}}{\Delta_{m}}\right)-{\left(\sum_{k=1}^{N}\frac{h_{m}C_{45}{}^{m}}{\Delta_{m}}\right)}^{2}$$

(36) $$\Delta_{m}=C_{44}{}^{m}C_{55}{}^{m}-{\left(C_{45}{}^{m}\right)}^{2}$$

These effective homogenized anisotropic elastic constants are then used in Section 3.1 for calculating the transmission coefficient from a layered medium into homogenized anisotropic media in which the SDH is embedded. These effective elastic constants are also used in Section 3.2 and Section 3.3 to calculate the group velocity, which is used to calculate the scattering of the SDH and also used to calculate the angle-dependent velocity for the TFM algorithm.

The next section describes the method to calculate the scattering from a side-drilled hole.

### 2.4. Scattering Coefficient of a SDH

Side-drilled holes are the reference reflectors, which are used in ultrasonic nondestructive testing [30]. As SDHs have a simple geometry, the exact scattering from the SDH can be calculated using the method of separation of variables [31]. The Kirchoff approximation could also be used to describe the scattering from an SDH, but it is a far-field and high-frequency approximation, where the size of the SDH is much larger than the wavelength of the inspecting wave. Many defects of importance, such as voids, porosity, etc., are smaller than the incident wavelength and hence Kirchoff scattering is not a good choice in such cases. Hence, for this study, the scattering coefficient is evaluated using the method of separation of variables, which is given in the below equations, where *A_scatt_*(ω) is the dimensionless scattering coefficient obtained by solving the scattering integral using the method of separation of variables, which is possible as the scatterer has a simple geometrical shape.

(37) $$A_{scatt}\left(\omega \right)=\frac{1}{L}\sqrt{\frac{2i}{\pi k_{p}}}\sum_{n=0}^{\infty }\left(2-\delta_{0n}\right)E_{n}\left(k_{\alpha }b\right)\cos\left(n\theta \right)e_{r}$$

(38) $$E_{n}=\frac{i}{2k_{p}b}\left\{1+\frac{I_{n}^{\left(2\right)}\left(k_{p}b\right)I_{n}^{\left(1\right)}\left(k_{s}b\right)-G_{n}^{\left(2\right)}\left(k_{p}b\right)G_{n}^{\left(1\right)}\left(k_{s}b\right)}{I_{n}^{\left(1\right)}\left(k_{p}b\right)I_{n}^{\left(1\right)}\left(k_{s}b\right)-G_{n}^{\left(1\right)}\left(k_{p}b\right)G_{n}^{\left(1\right)}\left(k_{s}b\right)}\right\}$$

(39) $$I_{n}^{\left(i\right)}\left(x\right)=\left(n^{2}+n-{\left(k_{s}b\right)}^{2}/2\right)H_{n}^{\left(i\right)}\left(x\right)-xH_{n-1}^{\left(i\right)}\left(x\right)$$

(40) $$G_{n}^{\left(i\right)}\left(x\right)=\left(n^{2}+n\right)H_{n}^{\left(i\right)}\left(x\right)-nxH_{n-1}^{\left(i\right)}\left(x\right)$$

where *H* is the Hankel function and *i =* 0, 1 corresponds to the order of the Hankel function, *L* is the length of the SDH, *θ* is the angle between the angle of incidence and angle of scattering, *b* is the radius of the SDH, δ is the Kronecker delta, and *e_r_* is the unit vector of the receiving transducer.

For the pulse echo response of an SDH embedded in anisotropic materials, Huang suggested that the scattering of the SDH is the same as that of an SDH embedded in an isotropic medium for a particular angle of incidence [32]. Hence, for a particular angle of incidence, we consider the equivalent homogeneous anisotropic medium as isotropic and calculate the properties at this particular angle of propagation.

The calculated scattering coefficient will be used in Section 3.2 to calculate the received signal from the SDH.

## 3. Development of a Model to Facilitate Scattering of SDH in a Layered Anisotropic Medium

This section provides the steps required to develop a model to simulate the scattering from an SDH that is embedded in a layered anisotropic medium and to post-process the FMC signals using model-assisted corrected TFM.

### 3.1. Reflection and Transmission Coefficients of Layered Structure Bounded by Anisotropic Media

In this section, we derive the equations for the reflection and transmission coefficients for a layered medium bounded by semi-infinite anisotropic media.

The reflection and transmission coefficients are derived by combining the transfer matrix method and the stiffness matrix method.

Consider the upper semi-infinite layer 0 as shown in Figure 1, where **A***^reflected^* is the amplitude of the wave reflected from the layered structure, and **A***^incident^* is the amplitude of the downward moving incident wave. Then, from Equation (5), we obtain

(41) $$\left[\begin{array}{l} u^{1}\left(0\right)\\ \sigma^{1}\left(0\right) \end{array}\right]=\left[\begin{array}{cc} D^{+} & D^{-}H^{-}\\ F^{+} & F^{-}H^{-} \end{array}\right]\left[\begin{array}{l} A^{incident}\\ A^{reflected} \end{array}\right]$$

**H** can be removed from the above equation as it controls the decay of the wave of complex wavenumbers in a finite thickness of the material and, as we are interested in only the semi-infinite layer, there is no decay due to this term.

(42) $$\left[\begin{array}{l} u^{1}\left(0\right)\\ \sigma^{1}\left(0\right) \end{array}\right]=\left[\begin{array}{cc} D^{+} & D^{-}\\ F^{+} & F^{-} \end{array}\right]\left[\begin{array}{l} A^{incident}\\ A^{reflected} \end{array}\right]$$

After matrix manipulation of Equation (41), we obtain the below equation:

(43) $$\left[\begin{array}{l} u^{1}\left(0\right)\\ \sigma^{1}\left(0\right) \end{array}\right]=\left[\begin{array}{c} D^{+}\\ F^{+} \end{array}\right]\left[A^{incident}\right]+\left[\begin{array}{c} D^{-}\\ F^{-} \end{array}\right]\left[A^{reflected}\right]$$

At the *N*^th^ layer, where *m = N* before the lower semi-infinite anisotropic medium, we have the following equation, as shown in Equation (6):

(44) $$\left[\begin{array}{l} u^{m}\left(h_{m}\right)\\ \sigma^{m}\left(h_{m}\right) \end{array}\right]=\left[\begin{array}{cc} D^{+}H^{+} & D^{-}\\ F^{+}H^{+} & F^{-} \end{array}\right]\left[\begin{array}{l} A^{transmitted}\\ A^{reflected} \end{array}\right]$$

There is no reflected upward traveling wave in the lower semi-infinite medium due to no reflection boundary being present, so

(45) $$A^{reflected}=0$$

Equation (44) can then be written as

(46) $$\left[\begin{array}{l} u^{m}\left(h_{m}\right)\\ \sigma^{m}\left(h_{m}\right) \end{array}\right]=\left[\begin{array}{c} D^{+}\\ F^{+} \end{array}\right]\left[A^{transmitted}\right]$$

We also know the stiffness matrix formulation given in Equation (15) as

(47) $$\left[\begin{array}{l} \sigma^{0}\left(0\right)\\ \sigma^{n}\left(h_{N}\right) \end{array}\right]=S\left[\begin{array}{l} u^{0}\left(0\right)\\ u^{n}\left(h_{N}\right) \end{array}\right]$$

We can rewrite and solve the above equations in terms of the incident, transmitted, and reflected amplitudes and the stiffness matrix as shown below:

(48) $$\left[\begin{array}{l} u^{1}\left(0\right)\\ u^{m}\left(h_{m}\right) \end{array}\right]=\left[\begin{array}{c} D^{+}\left(0\right)A^{incident}+D^{-}\left(0\right)A^{reflected}\\ D^{+}\left(n+1\right)A^{transmitted} \end{array}\right]$$

(49) $$\left[\begin{array}{l} \sigma^{1}\left(0\right)\\ \sigma^{m}\left(h_{m}\right) \end{array}\right]=\left[\begin{array}{c} F^{+}\left(0\right)A^{incident}+F^{-}\left(0\right)A^{reflected}\\ F^{+}\left(n+1\right)A^{transmitted} \end{array}\right]$$

(50) $$\left[\begin{array}{c} F^{+}\left(0\right)A^{incident}+F^{-}\left(0\right)A^{reflected}\\ F^{+}\left(n+1\right)A^{transmitted} \end{array}\right]=\left[\begin{array}{cc} S_{11} & S_{12}\\ S_{21} & S_{22} \end{array}\right]\left[\begin{array}{c} D^{+}\left(0\right)A^{incident}+D^{-}\left(0\right)A^{reflected}\\ D^{+}\left(n+1\right)A^{transmitted} \end{array}\right]$$

Carrying out matrix multiplication and rearranging the matrices in Equation (50), we formulate Equation (51) to calculate the amplitude of the reflected and transmitted wave in the semi-infinite medium:

(51) $$\left[\begin{array}{cc} -S_{11}D^{-}(0)+F^{-}\left(0\right) & -S_{12}D^{+}\left(n+1\right)\\ -S_{21}D^{-}\left(0\right) & -S_{22}D^{+}(n+1)+F^{+}\left(n+1\right) \end{array}\right]\left[\begin{array}{c} A^{reflected}\\ A^{transmitted} \end{array}\right]=\left[\begin{array}{c} S_{11}D^{+}(0)-F^{+}\left(0\right)\\ S_{21}D^{+}\left(0\right) \end{array}\right]\left[A^{incident}\right]$$

For simplicity, the amplitude of the incident wave is taken as unity and the above equation can then be solved to calculate the amplitude of the reflected and transmitted wave, which are the reflection and transmission coefficients, respectively, of the upper and lower bounding layers.

### 3.2. Calculation of the Scattering from SDH Embedded in the Medium

We use the bounded beam approach to calculate the received signal from an SDH as the SDH scattering has been calculated in the frequency–space domain and not in the frequency–wavenumber domain. In the bounded beam approach, the signal from the transmitting element to the scatterer, the signal received by the receiving element, and the scattering response of the SDH in the frequency–space domain are multiplied as shown below [33] in the frequency domain to produce the output signal, which is dimensionless.

(52) $$V(x_{t},x_{r},0,\omega )=V_{t}\left(x_{t},0,\omega \right)V_{r}\left(x_{r},0,\omega \right)A(x_{t},x_{r},\omega )$$

where

(53) $$V_{t}\left(x_{t},0,\omega \right)=\int_{-\infty }^{+\infty }v_{t}\left(k_{x_{t}},0\right)\beta \left(\omega \right)T\left(k_{x_{t}},0\right)e^{-i\left(k_{x_{t}}x_{t}\right)}dk_{x_{t}}$$

(54) $$V_{r}\left(x_{r},0,\omega \right)=\int_{-\infty }^{+\infty }v_{r}\left(k_{x_{r}},0\right)\beta \left(\omega \right)T\left(k_{x_{r}},0\right)e^{-i\left(k_{x_{r}}x_{r}\right)}dk_{x_{r}}$$

*T* is the transmission coefficient of the plane waves traveling from layered media into homogeneous equivalent anisotropic media, *β* is the system function [16,21], *v_t_* is the acoustic wavefield at the face of the transmitting transducer, and *v_r_* is the acoustic wavefield at the face of the receiving transducer calculated from the previous section. *A* is the scattering magnitude of the SDH calculated using Equation (37).

The received time domain signal from the scatterer is then calculated using

(55) $$V\left(x_{t},x_{r},t\right)=\int_{-\infty }^{+\infty }V(x_{t},x_{r},\omega )e^{-i\left(\omega t\right)}d\omega$$

Equation (55) gives the received FMC signal for scattering from a defect embedded in a medium. Equation (55) is used to generate the FMC data, which are used by the imaging algorithm to image the defect and the scattering from the defect.

The next section gives an explanation of the total focusing method.

### 3.3. TFM Imaging

The total focusing method is considered the gold standard of imaging algorithms [34,35]. It is a delay and sum algorithm that uses the entire full matrix capture data. The TFM algorithm generates an image by synthetically focusing on every pixel in the image domain, as given in the below equation:

(56) $$I(x,z)=\left|\sum V_{t,r}\left(\frac{\sqrt{{\left({\left(x_{1}\right)}_{t}-x_{1}\right)}^{2}+x_{3}{}^{2}}+\sqrt{{\left({\left(x_{1}\right)}_{r}-x_{1}\right)}^{2}+x_{3}{}^{2}}}{c}\right)\right|$$

where *I* is the intensity of the image at the point *x*,*z*, *c* is the velocity of the wave in the medium, and *V_t,r_* is the received signal for a transmitter receiver pair.

For anisotropic media, the velocity *c* is calculated using the Christoffel equation [17], which varies as per the angle of propagation. Hence, the varying group velocity in an anisotropic material is taken into consideration, which differentiates the model-assisted corrected TFM from the isotropic TFM.

### 3.4. Quantitative Comparison of the Images

The TFM image formed using experimental data is different from the one formed using the simulated data as the experimental TFM image additionally contains the scattering from the SDH and interaction of the signals with the layers below the SDH and backwall. In defect detection, the signal from the defect is important; hence, in order to compare the experimental and simulated images, and also for comparison between different simulated images, the SNR of the defect should be considered. In this context, the SNR is defined as the ratio of the peak amplitude of the scatterer to the noise in the image around the scatterer. The reverberations from the layers, and the signals from the laminated structure, are considered noise as they affect the scattering amplitude of the scatterer. In this case, the SNR is given by Equation (57):

(57) $$\mathrm{SNR}=\sqrt{\frac{\mathrm{Peak}\;\mathrm{Signal}\;(\mathrm{SDH})}{\sigma_{RMS}\left(\mathrm{Noise}\right)}}$$

The SNR for the simulated image can be calculated in the following steps:

1. Simulate the response from the embedded scatterer and calculate the peak amplitude of the scatterer.
2. Simulate the response of the laminate without the scatterer and calculate the root mean square of the amplitudes of the signal in a chosen region around the scatterer, which is the “noise” of the image.
3. Use Equation (57) to calculate the SNR of the SDH. The same procedure is carried out for the experimental TFM image, wherein the laminate FMC signals are processed before and after the SDH has been drilled into the laminate.

The next section presents the results for each step and the final image, which was simulated using TFM.

## 4. Simulation and Results

This section contains the simulation and experimental results for both homogeneous isotropic and anisotropic multilayered materials, followed by the Discussion section. The hardware used to acquire the experimental signals was the FI Toolbox from Diagnostic Sonar. The phased array transducers were from Olympus. The FI toolbox was the acquisition module, which acquired the signals and the signals were post-processed in MATLAB®. All computational work encompassing the modeling and implementation of the imaging algorithm was carried out using MATLAB 2017®.

The specifications of the transducers are shown in Table 1.

**Figure 2:**
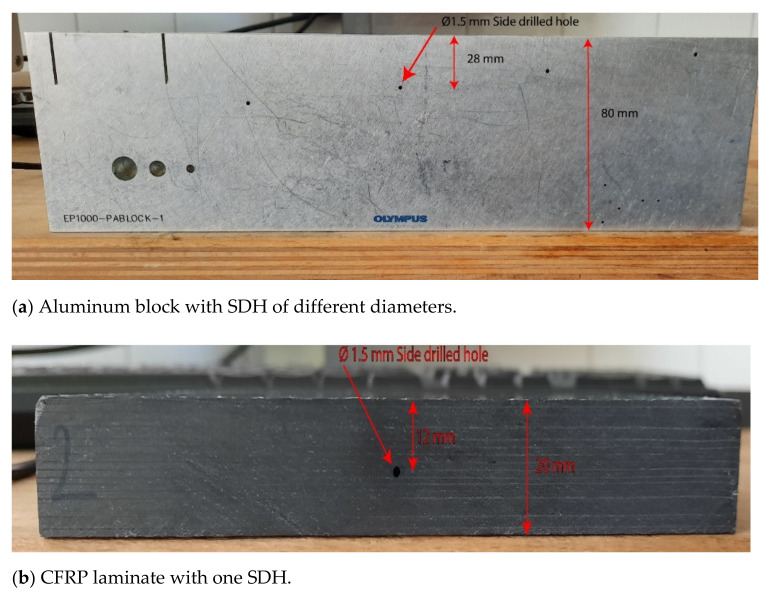
(**a**) Picture of the aluminum block with SDH used to verify the simulation results; (**b**) CFRP laminate with one side-drilled hole used to verify the simulation results.

For simulation and experimental purposes, we considered an 80 mm aluminum block (Olympus EP1000-PABLOCK-1) as shown in Figure 2a. For simplicity, only 1 SDH of diameter 1.5 mm at a depth of 28 mm was considered for simulation and experimental validation purposes. A CFRP laminate that was quasi-isotropic and 19 mm thick with (0/45/-45/90) layup was considered for simulation and experimental purposes and is shown in Figure 2b. There were 169 layers of UniDirectional CFRP prepreg of 110 μm thickness in the laminate, with a layer of epoxy resin with an approximate thickness of 5 μm between them. The laminate was manufactured from Toray TC380 unidirectional prepreg in an epoxy resin system. Manufacturing was carried out using autoclave curing. The SDH in the CFRP laminate was at a depth of 12 mm from the surface of the laminate and was manufactured by drilling. As the size of the SDH was relatively small and the SDH had a length of 20 mm, it was assumed that the delamination caused by drilling was minimal. For the purpose of the simulation, the layers containing the SDH and those below it were homogenized.

The aluminum and CFRP lamina properties were the same, as shown in Table 2. 

### 4.1. Calculation of Equivalent Homogeneous Properties

By substituting the lamina properties into Equations (21)–(35), we obtain the equivalent homogeneous anisotropic properties as shown Table 3.

The properties in Table 3 were then used to calculate the transmission coefficient of the plane waves into the semi-infinite anisotropic medium and were also used to calculate the group velocity in the medium.

In the next section, the simulation and experimental results for an SDH embedded in an isotropic medium are presented.

### 4.2. SDH Embedded in Aluminum Inspected by a 2.25 and 5 MHz Array

In this section, we present the simulation and experimental results of the scattering from an SDH embedded in an isotropic medium. Simulation and experimental FMC signals were generated for an isotropic material so as to prove the validity of the developed model for both isotropic and anisotropic materials. The images of an SDH embedded in an isotropic material were also generated to allow visual comparison of the differences between scattering in isotropic and anisotropic embedding media. 

**Figure 3:**
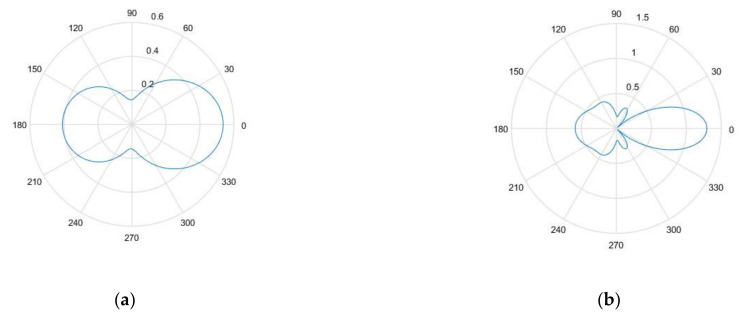
(**a**) Non-dimensional scattering magnitude of 1.5 mm diameter hole in aluminum at 2.25 MHz frequency; (**b**) non-dimensional scattering magnitude of 1.5 mm diameter hole in aluminum at 5 MHz frequency.

Figure 3a,b show the nondimensional scattering magnitude of the 1.5 mm diameter SDH embedded in aluminum when inspected by waves with a center frequency of 2.25 and 5 MHz, respectively. It was observed that as the frequency increased, the magnitude of the scattering amplitude also increased. It was also observed that as the wavelength of the inspecting wave increased as compared to the size of the SDH, the scattering became less directional.

**Figure 4:**
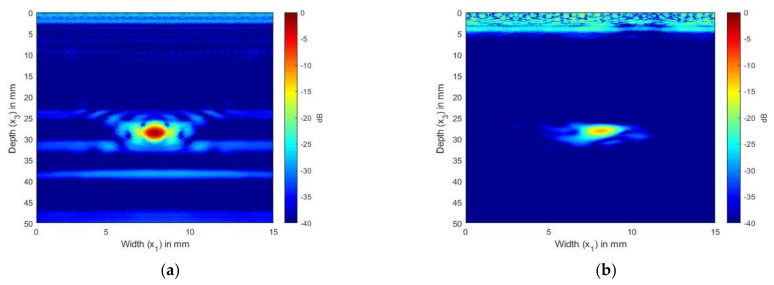
(**a**) TFM image of simulated SDH FMC signals in aluminum using the 2.25 MHz array; (**b**) TFM image of experimental SDH FMC signals in aluminum using the 2.25 MHz array.

In Figure 4a,b, we present the simulated and experimentally obtained TFM image of SDH embedded in aluminum. The aluminum block shown in Figure 2a was used to obtain the FMC signals experimentally.

Figure 4a,b show the scattering of an SDH of diameter 1.5 mm embedded in aluminum at a depth of 28 mm and inspected by an ultrasonic array with a frequency of 2.25 MHz. Figure 4a shows the TFM image generated from the FMC signals obtained from the simulation, whereas Figure 4b shows the TFM image generated for the FMC signals obtained experimentally.

**Figure 5:**
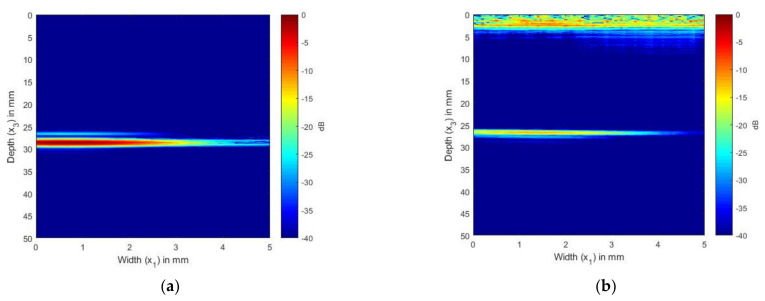
(**a**) TFM image of simulated SDH FMC signals in aluminum using the 5 MHz array; (**b**) TFM image of experimental SDH FMC signals in aluminum using the 5 MHz array.

Figure 5a,b show the scattering of an SDH of diameter 1.5 mm embedded in aluminum at a depth of 28 mm inspected by an ultrasonic array with a frequency of 5 MHz. Figure 5a shows the TFM image generated from the FMC signals obtained from the simulation, whereas Figure 5b shows the TFM image generated for the FMC signals obtained experimentally.

It can be seen from Figure 4a that the location and size of the SDH were accurate when the frequency of 2.25 MHz was used. The simulation results agreed qualitatively with the experimental results. When the SDH was inspected by the 5 MHz array, as shown in Figure 5, the SDH seemed to be spread over a large area. In the case of the 5 MHz array, the simulated and the experimental images were in good agreement. To enable a quantitative analysis between the simulation and experimental results, the SNR values of the SDH are provided in Table 4. The SNR values were calculated using the equation and procedure described in Section 3.4. It could be observed that the error between the SNR values was within the range of +/− 8 dB between the simulated and experimental values.

In the next section, the simulation and experimental results for an SDH embedded in an equivalent anisotropic medium are presented.

### 4.3. SDH Embedded in CFRP Inspected by Arrays with Center Frequencies of 2.25 and 5 MHz

**Figure 6:**
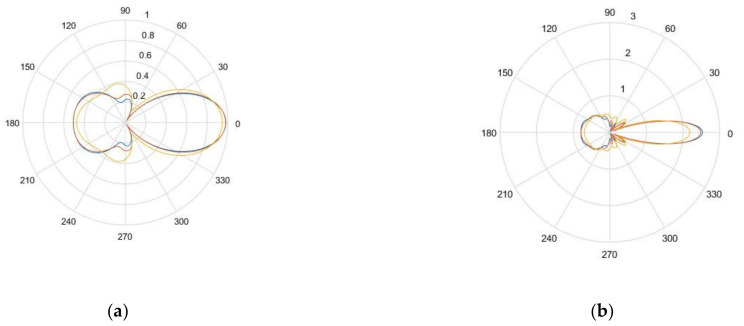
(**a**) Scattering amplitude as a function of angle of incidence for 0° (blue), 30° (orange), and 60 ° (yellow) for 1.5 mm diameter SDH embedded in homogenized CFRP at 2.25 MHz; (**b**) scattering amplitude as a function of angle of incidence for 0° (blue), 30° (orange), and 60 ° (yellow) for 1.5 mm diameter embedded in homogenized CFRP at 5 MHz.

Figure 6a,b give the nondimensional scattering magnitude of an SDH embedded in a homogenized CFRP laminate with equivalent homogeneous properties at center frequencies of 2.25 and 5 MHz, respectively. The scattering is given as a function of the scattering angle for angles of incidence of 0, 30, and 60 degrees. 

In Figure 6, it can be observed that as the angle of incidence increased, the scattering amplitude decreased and the directionality of the scattering was reduced. As observed in the case of the isotropic medium, as the frequency increased, the scattering magnitude increased.

**Figure 7:**
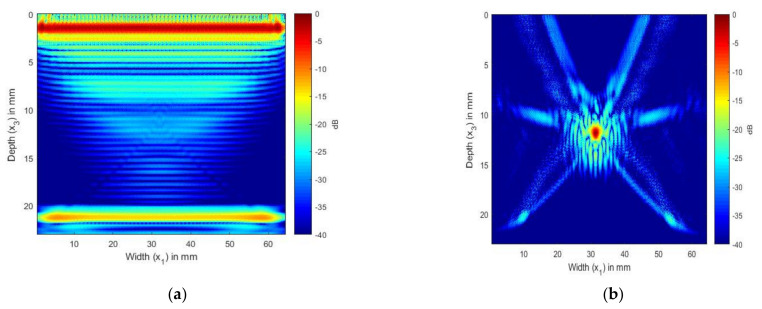
(**a**) TFM image of simulated FMC signals in CFRP laminate without SDH using 2.25 MHz array; (**b**) TFM image of simulated SDH FMC signals in equivalent homogeneous anisotropic laminate using 2.25 MHz array.

Figure 7 shows the TFM image generated from the simulated FMC signals for the laminate without an SDH and from the SDH embedded in an equivalent homogeneous anisotropic medium. Figure 7a shows the TFM image of the CFRP laminate without the SDH. The FMC signals were simulated using the model developed in a previous paper [16]. This image is the noise image as it shows the structural reverberations and internal reflections from the layers in the laminate, which contributed to the noise generated in the FMC signals. Figure 7b shows the TFM image of the scattering from the SDH generated from the FMC signals, which were simulated using Equation (55).

**Figure 8:**
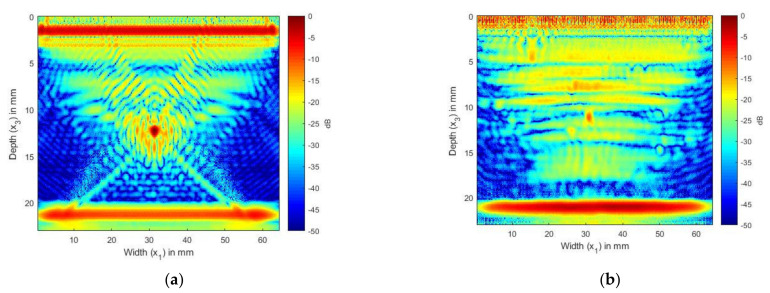
(**a**) Overlay image of simulated SDH signals with simulated composite laminate without SDH inspected by 2.25 MHz array; (**b**) TFM image of experimentally obtained FMC signals from CFRP laminate inspected by 2.25 MHz array.

In Figure 7a, the image of the CFRP laminate without the SDH, we can observe the internal reflections and reverberations from the layer interfaces. Figure 7b shows the scattering from the SDH embedded in a CFRP laminate. We can see that the SDH image is not exactly circular, it is spread across a diameter of 3 mm, and there is also lower-magnitude scattering around the SDH. To compare the images generated with the simulated FMC signals to the image generated using the experimentally obtained signals, we combined the signals obtained for Figure 7a,b to create Figure 8a. Figure 8a shows the image generated from the simulated FMC signals from the SDH and laminate and Figure 8b shows the image generated from the experimentally obtained FMC signals. It can be observed that Figure 8a is in quite good agreement with Figure 8b, with the noise seen in Figure 7b contributing to the noise in the composite image. Figure 8b shows more noise than Figure 8a and the source of the noise could be the manufacturing process, including the varying thickness of plies and epoxy after manufacture, which is difficult to account for in a simulation.

**Figure 9:**
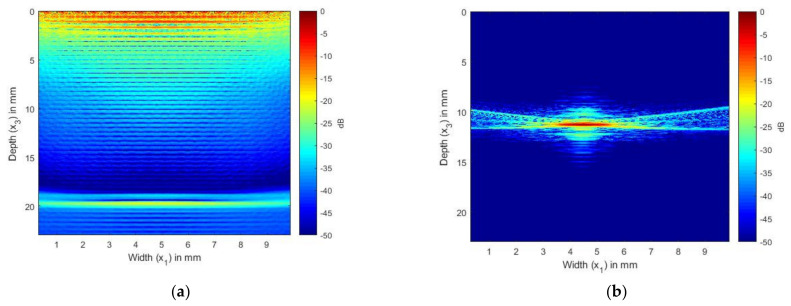
(**a**) TFM image of simulated FMC signals in CFRP laminate without SDH using the 5 MHz array; (**b**) TFM image of simulated SDH FMC signals in equivalent homogeneous anisotropic laminate using the 5 MHz array.

**Figure 10:**
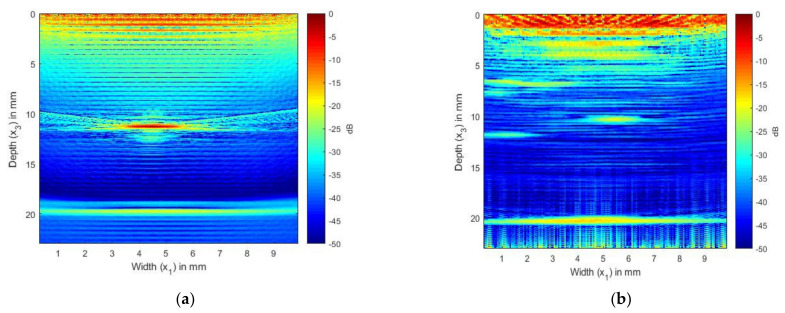
(**a**) Overlay image of simulated SDH signals with simulated composite laminate without SDH inspected by a 5 MHz array; (**b**) TFM image of experimentally obtained FMC signals from CFRP laminate inspected by a 5 MHz array.

In Figure 9 and Figure 10, we present the results of the simulation carried out using Array 2 with a central frequency of 5 MHz. In Figure 9a, we can see the internal reflections and reverberations of the plies, which are more pronounced than those in Figure 7a. Figure 9b shows the SDH at a depth of 12 mm. As in the isotropic case, the SDH appears to be spread over a large area, with noise at the edges of the SDH. Figure 10a shows the image generated from the simulated FMC signals from the SDH and laminate, and Figure 10b shows the image generated from the experimentally obtained FMC signals. It can be observed that Figure 10a is in good agreement with Figure 10b, with the noise seen in Figure 10b contributing to the noise in the composite image. We can observe more noise and artifacts in Figure 10b, which could be due to manufacturing inconsistencies. 

To enable a quantitative comparison between the experimental and the simulation results, Table 5 shows the SNR of the simulation and experiment.

An error in the range of 14 dB to 18 dB can be observed from Table 5. The error is higher in the case of the CFRP, for various reasons, such as the absence of the effect of layering below the SDH and the backwall reflections on the amplitude of the SDH signal. As the SDH is of a small diameter, the effects of the layers just below the SDH and the layers in which the SDH is embedded on the received amplitude are higher than in the simulations. It can also be seen that defects during manufacture also influence the signal from the SDH, which cannot be included beforehand in the simulation.

## 5. Discussion

Figure 4 and Figure 5 show the comparison between the simulated and the experimental TFM images of SDH embedded in an aluminum block. It was observed that when the size of the SDH was larger than the wavelength of the inspecting wave, the SDH appeared to spread over a larger area, as in the case of the 5 MHz array. This is because the scattering at these higher frequencies was of a higher magnitude, and the decrease in the scattering magnitude with the scattered angle was less, as observed in Figure 3. A quantitative comparison of the SNR also led to the conclusion that the simulation images for defects in isotropic media agreed well with the experimental images. 

**Figure 11:**
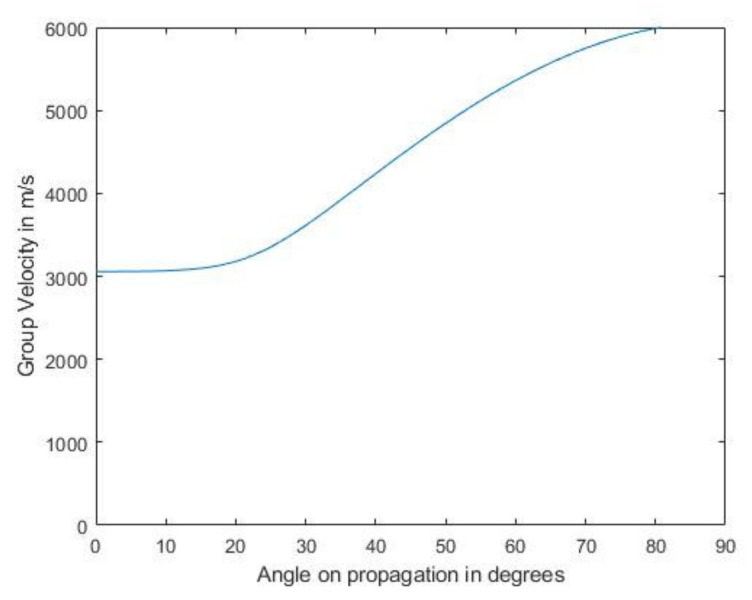
Group velocity of the longitudinal wave for different angles of propagation.

Next, Figure 8 and Figure 10 present a comparison between the simulated and experimental TFM images of SDH embedded in a CFRP laminate. Here, the layer in which the SDH is embedded and the layers below it were modeled as a semi-infinite anisotropic region using the equivalent homogeneous anisotropic properties given in Table 3. As in the case of an isotropic embedding medium, the SDH at 2.25 MHz showed good agreement between the simulated and experimental image. More noise was visible around the SDH. This noise was due to the anisotropic velocity in different directions and also due to the creeping wave [36]. As the pitch between the elements was 1 mm and the array was a 64-element array, the angles of incidences were large, and the theoretical group velocities, as shown in Figure 11, along these angles were large, leading to the noise accompanying the scattering signal. The group velocity was calculated using the expression given in Equation (58), where *u_p_* is the group velocity, *c_p_* is the phase velocity, *c_ijkl_* is the elastic constant, *p* is the polarization direction, and *n* is the unit vector in the direction of propagation of the wave:

(58) $$u_{pi}=\frac{c_{ijkl}n_{k}p_{l}p_{k}}{\rho c_{p}}$$

Figure 9 shows the scattering from an SDH when inspected with Array 2, which had a central frequency of 5 MHz. Less noise was observed around the edges as compared to the TFM image using a 2.25 MHz array. This was due to the fact that, because of the smaller pitch and lower number of elements in the array, the maximum angle of propagation was confined to less than 40° and hence the variation in the group velocity was not very large.

In CFRP, the image of the SDH was elliptical due to the various effects of the diffraction of the layers from above, the inspecting wavelength as compared to the size of the SDH, and the anisotropic velocity. The simulation provided a good tool to determine which frequencies need to be used to inspect a certain material, SDH size, location, etc. We observed that the difference in the SNR values between the simulation and experimental images was larger for CFRP as compared to aluminum. One of the reasons for this is that the SDH was assumed to be embedded in a homogeneous medium and the layers beneath it were not taken into account in the simulation. The layers below the SDH will also contribute to the noise in the image and influence the magnitude of the SDH, hence reducing the SNR in the experimental TFM image.

## 6. Conclusions

This paper proposes a modeling technique based on the Gaussian beam and the recursive stiffness matrix method to simulate the scattering from an SDH embedded in a CFRP laminate. The simulation requires the integration of different modules to simulate the scattering of an SDH. A novel method is implemented to calculate the transmission and reflection coefficients from layered media into a semi-infinite anisotropic medium by combining the transfer matrix and recursive stiffness matrix approaches.

The modeling technique takes into consideration the diffraction, anisotropic velocity, and inspection frequency effects while simulating the scattering from the SDH embedded in a layered medium. The simulation and the experimental results are in good agreement, which was observed qualitatively using TFM to image the FMC signals and also quantitatively by comparing the SNR values for both isotropic and anisotropic samples. To the best of the authors’ knowledge, there are no analytical models that can be used both in immersion and contact setups based on multi-Gaussian beams and the stiffness matrix method to simulate the scattering from SDHs. Hence, this paper provides a model that can be used both in immersion and contact setups and is both computationally inexpensive and accurate. Future work would include a full quantitative comparison, with a well-defined sample that has been validated using CT, and the modeling and validation of different defects, such as porosity and delaminations in plane and curved composite structures.

## Figures and Tables

**Table 1:** Transducer array specifications [11].

	Centre Frequency (MHz)	Pitch (mm)	Number of Elements
Array 1	2.25	1	64
Array 2	5	0.6	16

**Table 2:** Material properties [35].

Properties	Aluminum (GPa)	Carbon/Epoxy >65% Fiber-Volume Fraction (GPa)
C11	110	13.89(1+0.02i)
C22	110	13.89(1+0.02i)
C33	110	121.7(1+0.001i)
C12=C21	60	6.43(1+0.011i)
C13=C31	60	5.5(1+0.007i)
C23=C32	60	5.5(1+0.007i)
C44	25	5.1(1+0.066i)
C55	25	5.1(1+0.066i)
C66	25	3.73(1+0.027i)

**Table 3:** Equivalent homogeneous anisotropic properties.

Properties	Values in GPa
$\bar{C_{11}}$	54.76(1+0.002i)
$\bar{C_{22}}$	54.76(1+0.002i)
$\bar{C_{33}}$	13.89(1+0.02i)
$\bar{C_{12}}$	18.53(1+0.01i)
$\bar{C_{13}}$	5.96(1+0.004i)
$\bar{C_{23}}$	5.96(1+0.005i)
$\bar{C_{44}}$	4.3(1+0.06i)
$\bar{C_{55}}$	4.3(1+0.06i)
$\bar{C_{66}}$	18.12(1+0.03i)

**Table 4:** SNR of SDH in aluminum.

Central Frequency of Array	SNR of SDH in Simulated Image	SNR of SDH in Experimental Image	Difference
2.25 MHz	−42.9 dB	−39.5 dB	−3.4 dB
5 MHz	−26.1 dB	−33.4 dB	−7.3 dB

**Table 5:** SNR of SDH in CFRP.

Central Frequency of Array	SNR of SDH in Simulated Image	SNR of SDH in Experimental Image	Difference
2.25 MHz	−42.9 dB	−25.6 dB	−17.3 dB
5 MHz	−35.48 dB	−21 dB	−14.48 dB

## Data Availability

Not applicable.
